# Pulmonary infections in the returned traveller

**DOI:** 10.1186/s41479-017-0026-1

**Published:** 2017-01-25

**Authors:** Ashleigh Trimble, V. Moffat, A. M. Collins

**Affiliations:** 1 Crosshouse Hospital, Kilmarnock Road, Crosshouse, KA2 0BE UK; 2 Respiratory Infection Group, Liverpool School of Tropical Medicine, Pembroke Place, Liverpool, L3 5QA UK; 3 Aintree Hospital, Longmoor Lane, Liverpool, L9 7AL UK; 4 Respiratory Research Group, Royal Liverpool and Broadgreen University Hospital Trust, Prescot Street, Liverpool, L7 8XP UK

**Keywords:** Travel, Traveller, Pulmonary infections, Tropics, Respiratory infections

## Abstract

Pulmonary infections in the returned traveller are a common presentation. A wide variety of infections may present with pulmonary symptoms. It is important for clinicians to differentiate the cause of these symptoms. The risk of contracting certain travel-related pulmonary diseases depends on travel destination, length of stay, activities undertaken and co-morbidities. Some pathogens are found worldwide, whilst others are related to specific locations. This review article will discuss the approach to diagnosing and treating pulmonary infections in the returned traveller.

## Background

International travel has steadily increased over the last few decades, and is no longer only for the wealthy and healthy. The number of travellers reached more than one billion for the first time in 2012 and continues to rise [1]. There are many risk factors that increase the likelihood of developing a travel-related illness and this review will focus on pulmonary-related infections (Table 1) [2–4]. Many illnesses are self-limiting and are a result of common pulmonary viruses that are prevalent worldwide [3, 5] but in certain circumstances travel-related pulmonary infections are serious and have potentially fatal implications, and can even cause epidemics.

**Table 1:** Specific Risk factors

Risk activities	Common	Occasional
Bites		
Tick		Q fever
Mosquitos	Tropical eosinophilia *(Wuchereia bancrofti* and *Brugia malayi)*	Dirofilarisis
Animal	Avian influenza, Nipah virus, Hantaan virus, MERS	Q fever, tularemia, plague, SARS
Dust exposure (e.g., caves or deserts)	Coccidioimycosis, histoplasmosis	
Cruise ships/resorts	Legionella	
Rural/Farm	Q fever	
Soil	Strongyloidiasis, hookworm, ascariasis	Melioidosis
Fresh-water exposure	Schistosomiasis, leptospirosis	
Ingestion		
Undercooked/raw food		Paragonimus, toxocariasis, hydatidosis
Sexual exposure	HIV	
Host factors		
Immunocompromised	Tuberculosis	

*SARS* severe acute respiratory syndrome, *MERS* Middle Eastern respiratory syndrome, *HIV* human immunodeficiency virus

Air travel has shown how efficiently pulmonary infections can be transmitted. Data suggests the risk of disease transmission to a symptom-free passenger within the aircraft cabin is associated with sitting within 2 rows of a contagious passenger for a flight time of more than 8 h. This association is mainly derived from investigations of in-flight transmission of tuberculosis (TB) [6]. Aircraft cabin ventilation appears to be a key factor enabling disease transmission. This was highlighted by the spread of severe acute respiratory syndrome (SARS) during a short-haul flight in 2002 [5]. With sea travel (especially cruising) becoming more popular, the ageing population of travellers with multiple pre-existing conditions is also a concern.

Clinicians should be aware of particular diseases that may present in the returned traveller and rank their diagnostic likelihood based on destination, length of stay and symptom duration (Tables 2 and 3).

**Table 2:** Incubation periods of pulmonary infections

Incubation Period	Infection
Short (<10 days)	Viral: influenza, SARS, MERS, Nipah Bacterial: common organisms causing pneumonia (*Streptococcus pneumoniae*, *Haemophilus influenzae*), melioidosis, Legionellosis, plague, pertussis, diphtheria Fungal: histoplasmosis
Medium (10–21 days)	Viral: MERS, Nipah, hantavirus Bacterial: pertussis, melioidosis Fungal: histoplasmosis, coccidioidomycosis Eosinophilic: ascariasis, hookworm, strongyloides, toxocariasis
Long (>21 days)	Bacterial: Q fever, TB Eosinophilic: schistosomiasis, hydatidosis, dirofilariasis, paragonomiasis, *Wuchereia bancrofti, Brugia malayi*

*SARS* severe acute respiratory syndrome, *MERS* Middle Eastern respiratory syndrome, *TB* tuberculosis

**Table 3:** Causes of pulmonary infections associated with specific geographical areas

Geographical Area	Common	Occasional	Rare but important
Sub-Saharan Africa	HIV-associated pulmonary infections, TB	Katayama syndrome, tropical pulmonary eosinophilia (*Wuchereia bancrofti*), Loeffler’s syndrome (hookworm, strongyloides) hydatidosis	Histoplasmosis, pneumonic plague, paragonomiasis
North Africa, Middle East and Mediterranean		Q fever, tropical pulmonary eosinophilia (*Wuchereia bancrofti)* Loeffler’s syndrome (hookworm, ascariasis, strongyloides) hydatidosis, toxocariasis	MERS
Eastern Europe and Scandinavia	Legionellosis	Pertussis, toxocariasis	Hantavirus, tularemia
South and Central Asia	diphtheria, TB, avian flu	Melioidosis, Loeffler’s syndrome (hookworm, ascariasis, strongyloides) hydatidosis	Nipah virus
Southeast Asia	avian flu	Melioidosis, tropical pulmonary eosinophilia (*Brugia malayi*), Loeffler’s syndrome (strongyloides, hookworm), leptospirosis, diphtheria	SARS, hantavirus, Nipah virus, dirofilariasis, Katayama syndrome, paragonomiasis
Northern Australia		Q fever	Melioidosis, dirofilarisis, pertussis
Latin America and Caribbean		Coccidioidomycosis histoplasmosis, leptospirosis, diphtheria, tropical pulmonary eosinophilia (*Wuchereia bancrofti*), Loeffler’s syndrome (ascariasis, strongyloides), hydatidosis, toxocariasis	Hantavirus, dirofilariasis
North America	Legionellosis, pertussis, diphtheria	Coccidioidomycosis histoplasmosis, toxocariasis	Dirofilariasis, Q fever

*HIV* human immunodeficiency virus, *SARS* severe acute respiratory syndrome, *MERS* Middle Eastern respiratory syndrome, *TB* tuberculosis

Pneumonia is a frequent cause of fever amongst returned travellers [7]. Awareness of travel locations may be key to identifying the organism responsible, and polymerase chain reaction (PCR) testing is very important to look for atypical etiologies. Routinely, patients are started on antibiotics based on local guidelines for community-acquired pneumonia. It is unlikely these regimens will contain appropriate first-line therapy or be of sufficient duration to cover for pneumonias caused by *Coxiella burnettii* (Q fever) [8] or *Burkholderia pseudomallei* (melioidosis), which have been isolated in recent outbreaks that have occurred in Europe and South East Asia [9].

Pulmonary infections are diagnosed in up to 24% of returned travellers with fever, making it as common as travellers’ diarrhea, with influenza being the most common vaccine-preventable infection acquired (vaccine protective against H1N1, H3N2 and two influenza B virus strains) [10, 11]. Pre-travel advice should be that travellers with co-morbidities should be vaccinated against both seasonal influenza virus and pneumococcus [12]. It should be noted that the influenza vaccine does not currently offer protection against the most recent strains causing epidemics (H5N1, H7N9) and the risk of acquiring the illness depends on season of travel and length of stay in affected areas [13].

In this review, we will discuss various pulmonary infections that are more common in returned travellers. A clear and thorough approach to a symptomatic returned traveller is an essential skill for the acute/general physician and general practitioner (Table 4). Particular attention should be given to infections that may spread by human-to-human transmission. Clinicians should be aware of where to source information should they suspect a travel-related infection or if they have concerns for public health. Infections related to travel are constantly evolving and it is important for clinicians to remain up to date with current disease outbreaks and management recommendations.

**Table 4:** Checklist for taking a travel history

Questions	Examples
Country of travel and accommodation	Latent diseases, possible exposures
Occupation, hobbies, activities	Healthcare worker, business, visiting relatives, farmer, cave explorer, water exposure
Prophylaxis	Immunizations, prophylaxis, insect repellents
Treatment or procedures	Hospital treatment in country, tattoos
Diet	Seafood, raw food
Sex	Unprotected sex, paid sex, multiple partners
Animals	Birds, domestic animals, wildlife

## Pulmonary infections caused by viruses and bacteria

Upper pulmonary infections are more common than lower pulmonary infections. In general, the types of pulmonary infections that affect travellers are similar to those in non-travellers, and exotic causes are rare [2, 13, 14]. An Australian study collected data over a 3-year period; it showed around 28% of Australian travellers returning from Asia developed an acute pulmonary infection within 72 h (defined as an illness episode involving the presence of at least two upper pulmonary symptoms; e.g., sore-throat, coryza). This translates to an incidence of 106.4 per 10,000 traveller days. PCR testing on these travellers was analyzed for influenza A and B virus, adenoviruses, respiratory syncytial virus (RSV), picornaviruses and parainfluenza viruses 1, 2, and 3. Only 1% of these travellers acquired influenza A virus, with no data for the other detectable viruses included [15].

There is evidence that contracting a common upper pulmonary virus potentiates the risk of contracting a tropical disease as well. A study in 2008 [16] showed that during an epidemic of chikungunya in India, 87% of confirmed cases were also co-infected with RSV and another upper respiratory virus, 41% were co-infected with RSV alone and 9% were co-infected with influenza virus and adenovirus. It is likely that a parallel outbreak of pulmonary viral infections during this time resulted in higher morbidity and mortality during this epidemic.

Bacterial pathogens are generally not related directly to a particular travel destination. *Streptococcus pneumoniae, Mycoplasma pneumoniae, Haemophilus influenzae,* and *Chlamydophila pneumoniae* are the most common organisms known to cause pneumonia throughout the world and risk of infection is not directly related to travel. More specific illnesses caused by *C. burnettii* (Q fever), *Legionella pneumophila* (Legionella), *Bordetella Pertussis* (pertussis), *Corynebacterium diphtheriae* (diphtheria) and *Leptospira* (leptospirosis) are normally a result of an epidemic within a country [2, 10, 14]. This list may be very relevant to the treating clinician.

Travel to international mass gatherings is a proven risk factor for the spread of pulmonary infection. For example, during pilgrimage to Mecca, pneumonia caused by *S. pneumoniae* is the leading cause of hospitalization and intensive care unit admissions during the Hajj (nearly 40% of patients). Out of 300 participants less than 5% had been vaccinated despite 65% being eligible. Improved pre-travel vaccination advice could prevent diseases for many travellers [12].

### Influenza

Particular attention should be paid to travellers returning from influenza-endemic areas, taking into account newly reported sub-types. The burden of influenza occurs in the winter months in temperate regions, in the Northern Hemisphere from November to April and the Southern Hemisphere from April to October. In the tropics, the virus circulates at a low level all year round [17]. Influenza is diagnosed from viral throat swabs using real time PCR (rt-PCR) that can differentiate particular influenza subtypes [18].

Avian influenza sub-type H5N1 virus has caused death in humans since 1997, predominately in Asia and North East Africa, with a current upsurge of cases in Egypt. From 2003 to October 2015, there have been a total of 844 laboratory-confirmed cases with mortality rates of more than 50% [19]. H7N9 is the most recently reported sub-type. It was first diagnosed in March 2013 in China and is characterized by rapidly progressive pneumonia, pulmonary failure and acute pulmonary distress syndrome (ARDS) [18]. As of October 2015, there have been 679 laboratory-confirmed cases, with 275 reported deaths [19].

Treatment of influenza remains a controversial topic but generally supportive management is adopted. Current recommendations are early administration of a neuraminidase inhibitor (e.g., oseltamivir) to the affected individual and their close contacts [18]. A recent meta-analysis of the H1N1 pandemic showed that hospitalized patients treated with a neuraminidase inhibitor had a lower mortality, and that this was further reduced by early instigation [20].

Prevention of avian influenza has proved difficult due to the antigenic diversity of the circulating viruses, which has hampered vaccine development [21]. Healthcare professionals should take post-exposure prophylaxis (e.g., oseltamivir) after a patient has been diagnosed with avian influenza and personal protective equipment is highly recommended.

### Severe acute respiratory syndrome (SARS) and Middle Eastern respiratory syndrome (MERS)

Like influenza, other viral epidemics are gathering publicity, heightening awareness and recognition worldwide. MERS and SARS are zoonotic coronavirus infections with regular media coverage due to their symptom severity, associated mortality rates and anxiety regarding potential human-to-human transmission. Bats are thought to be the main intermediate host for both viruses, while there is compelling evidence that camels are also responsible for transmission of the MERS coronavirus [22, 23].

SARS was first reported as an atypical pneumonia to the World Health Organization (WHO) in November 2002 in China and subsequently spread worldwide. The first reported epidemic occurred in a Hong Kong hotel in February 2003. The virus was transmitted to 16 hotel guests who were staying on the same floor as an infected individual, and the virus spread through international air travel thereafter. WHO identified the coronavirus responsible through nasopharyngeal culture within 2 weeks of this event. More than 30 countries reported affected individuals, with more than 8,000 confirmed cases worldwide and a 9.6% mortality rate [24].

The pulmonary symptoms associated with SARS (typically, non-productive cough and subsequent dyspnea) did not present until several days after initial exposure to the virus. As SARS is caused by an airborne virus, the risk of transmission was greatest amongst those involved in the direct care of an infected individual. Since no medications effectively treated SARS, interrupting transmission was the key to controlling the outbreak. Simple public health measures, such as wearing surgical masks, offered the most effective protection against transmission and there were no further confirmed cases as of July 2003 [24].

The public health lessons learned during the SARS epidemic have proved invaluable in limiting the spread of MERS cases worldwide. MERS was first recognized in September 2012 and, as of October 2015, 1,611 cases have been reported with more than 570 related deaths [25]. To date, the largest cluster of cases to occur outside of the Middle East was reported in South Korea in May 2015. The index case was a businessman who developed pulmonary symptoms upon returning from a trip to the Middle East. A total of 185 cases were identified, with 36 deaths (19.5% mortality rate) [26]. This proved the migrating capabilities of the virus and potential for outbreaks worldwide. Any clinician who sees an individual returning from the Middle East presenting with fever and pulmonary symptoms within 14 days of travel should consider MERS as a diagnosis and prompt investigations (diagnosed by nasopharyngeal culture) and public health control methods should be initiated. No specific treatments or vaccine have been developed to date.

As with most infections, the groups with the poorest outcomes related to MERS infection are the young (<12 years), the elderly (>65 years), immunocompromised patients and those with multiple co-morbidities [22]. Clinically, presentation is similar to SARS but MERS has a longer incubation period and greater severity of pulmonary symptoms and an even higher mortality risk. All cases of MERS confirmed worldwide to date have had a direct or indirect link with the Middle East—predominately Saudi Arabia. It has proven to be less contagious than SARS so far, although it is expected that a more virulent virus will evolve.

### The plague

Where SARS and MERS have attracted much media coverage, it should not be forgotten that the plague bacillus (*Yersinia pestis*) still affects a great number of people each year [27]. The pneumonic plague has seen endemic outbreaks in certain parts of Africa, with sporadic cases occurring worldwide.

Historically, there have been 3 pandemics involving the bubonic (swollen, painful lymph nodes) and pneumonic plague in the 6th century, the 14th century and the 19th century; however, it also re-emerged in the 1990s with a more limited distribution [27].

The pneumonic plague occurs as a complication of the bubonic plague and is the only form capable of transmission from human-to-human via pulmonary droplets. Clinical sequelae are a dry cough within 24 h of first exposure, which becomes productive before hemoptysis occurs with the sputum becoming increasingly more bloodied within the next 24–48 h—this is a sign of certain mortality. The major clinical clues in diagnosing the pneumonic plague are travel history, rapid onset of symptoms (sometimes hours from being asymptomatic to fever and cough/hemoptysis) and bilateral pneumonia [27, 28]. The patient is most contagious when hemoptysis develops; simple protective measures, such as wearing masks, help prevent transmission [28].

The last reported outbreak of the pneumonic plague was in Madagascar in September 2014. There were 263 reported cases, with a 26.9% mortality rate. The plague has been endemic on the island for the past 30 years—it is the most severely affected country in the world [29].

While developing countries are at most risk of outbreaks of the disease, sporadic cases can occur elsewhere. Four patients were diagnosed in July 2014 with pneumonic plague in Colorado, United States of America (USA). The index patient contracted the disease from his pet dog. No deaths occurred and all patients rapidly responded to antibiotic treatment; streptomycin, tetracyclines and sulphonamides are used as the standard antibiotics, with gentamicin and fluoroquinolones as alternatives [30]. All close contacts should be given prophylactic antibiotics and monitored for symptoms.

The plague bacillus has previously been used in biological warfare during the 20th century. *Yersinia Pestis* is an attractive agent due to easy accessibility, simple culture needs and transmission via aerosol [31]. It is important point to note that a plague outbreak can be easily controlled with simple public health measures and readily available antibiotics; however, if inappropriately managed the pneumonic plague is almost certainly fatal.

### Hantaan and Nipah viruses

Two viral infections noted to be causing outbreaks throughout the world are the Hantaan (causing hantavirus cardiopulmonary syndrome [HPS]) and Nipah viruses. Both diseases were microscopically discovered in the 1990s and are zoonotic; they are transmitted to humans via rodents and via bats and pigs, respectively. Both have a range of clinical symptoms and may present with flu-like symptoms continuing onto encephalitis with Nipah virus or pulmonary failure and cardiogenic shock in Hantavirus-related infections [32, 33].

The first Nipah outbreak was in Malaysia in 1999; a total of 276 cases were diagnosed with a 38.4% mortality rate [34]. The outbreak spread into Singapore and it was discovered that the virus was transmitted to humans from pigs; this resulted in a cull of more than 1 million pigs to control the outbreak. Since then, outbreaks have occurred in Bangladesh and India with a mortality rate of around 75% [35]. Most cases presented with pulmonary symptoms (cough, dyspnea and bilateral opacities covering the majority of the lung fields on chest radiograph) and there was a higher human-to-human transmission rate in comparison to the Malaysian outbreak [36]. The Nipah virus was readily identified in the saliva of infected patients, allowing easy disease transmission.

There have only been outbreaks of the Nipah virus in South East Asia to date, but there is potential for this disease to occur in other countries. The intermediate host (fruit bats from the *Pteropodidae* family) has an extensive habitat distribution and is native to parts of Africa and northern Australia [37]. The treatment is limited to supportive care—control methods to prevent human-to-human transmission are very important to stop rapid spread. Studies have shown the antiviral drug ribavirin may reduce mortality from the infection [33].

At present, more than 21 Hantaviruses from the *Bunyaviridae* family have been identified as causing illness in humans. Signs of infection range from proteinuria to pulmonary edema and frank hemorrhagic illnesses [32]. HPS is most likely to occur in the Americas although there are sporadic case reports from Europe [38]. The disease is most likely to be transmitted in areas where sanitation is poor or in rural farming areas. Rodents shed the virus in their urine, droppings and saliva, and the virus is transmitted between humans via pulmonary droplets [32].

The first reported outbreak occurred in 1993 in the USA, although retrospectively it seems the Hantaviruses have been causing fatal pulmonary illnesses since the 1950s [39]. The cluster of around 30 cases in 1993 occurred in an area of southwest USA known as the ‘Four Corners’. Clinicians identified a series of cases of a severe and acute pulmonary illness affecting young healthy adults where non-cardiogenic pulmonary edema developed rapidly. To date, more than 600 cases of HPS have been reported in the USA [40]. In 2012, the virus was discovered at Yosemite National Park where potentially hundreds of travellers were exposed to the infection. Ten cases of HPS were confirmed, 3 of which were fatal [41].

Treatment for Hantavirus remains supportive. Intravenous fluids should be used with caution, as aggressive fluid resuscitation is likely to accelerate the progression to pulmonary edema and failure due to capillary leakage [32]. Extracorporeal membrane oxygenation (ECMO) treatment has dramatically reduced mortality rates [42]. The benefits of ribavirin therapy remain undetermined due to small trial numbers [43]. To date, only one Hantavirus has evolved to transmit via human-to-human contact (the Andes virus, named after its geographical discovery location in 1996).

Accurate diagnosis requires a high degree of suspicion and should be considered in an individual presenting from a recent stay in North or South America with rapidly progressing pulmonary symptoms and/or signs [32]. The risk to a traveller in contracting any of the above outbreak diseases remains small given their limited geographical distribution at present. The risk of exposure may be increased by several factors (Table 1) such as activities undertaken, close contact with an infected individual, and not seeking appropriate pre-travel information or recommendations. As a clinician it is important to have up-to-date knowledge on current outbreaks occurring worldwide and their relevant geography as prompt recognition and diagnosis may prevent an epidemic.

### Q fever

Q fever, caused by *C. burnettii*, is a worldwide zoonotic disease. Outbreaks in humans have been linked to abattoirs and carriage of *C. burnettii* by wind from farms of affected animals. Ticks can also act as a reservoir. It is not strictly a travel-related illness but it is most likely found in areas where contamination with animal waste is common. It presents as an acute febrile illness with non-specific signs such as atypical pneumonia [8]. Rural travelling is the greatest risk factor for acquiring the disease. Most recently, Q fever outbreaks have occurred in Hungary, where 70 cases were confirmed by micro-immunofluorescence testing and treated with 3 weeks of a tetracycline. No deaths occurred. Efforts to reduce the spread of Q fever after an outbreak include elimination of manure and disinfection of affected farms [8].

### Legionella

Legionella (*L. pneumophila)* is a bacterium that can cause anything from a mild febrile illness to the potentially fatal form of pneumonia known as Legionnaires’ disease. The bacterium is ubiquitous in the environment, and the same agent causes sporadic cases and outbreaks. Outbreaks are typically linked to contaminated water systems where conditions are ideal for rapid growth of the organism [44]. In 2014, the WHO confirmed 302 cases of Legionellosis in Lisbon, Portugal, that were related to a contaminated industrial cooling tower, with 5 deaths as a result of the disease [45]. Most cases are reported after travel to developed countries in Europe or North America. At risk individuals are those with co-morbidities, the elderly, smokers and the immunosuppressed. It is caused by inhalation of aerosols containing the bacterium and accounts for 2–15% of hospital admissions for community-acquired pneumonia, with a summer or autumn peak [45]. Identification of affected individuals is by urinary PCR and treatment is with a macrolide antibiotic [46].

### Pertussis and diphtheria

Pertussis and diphtheria are vaccine-preventable pulmonary infections; poor vaccine uptake has led to a recent increase in cases in developed countries. It is important, therefore, to take a full vaccination history in returned travellers who present with pulmonary symptoms [47].

Pertussis is a worldwide endemic-epidemic disease with outbreaks most likely during summer/autumn time. Pertussis is thought to still cause around 63,000 deaths per year in children under 5 years of age, although there is uncertainty over these estimates in view of the paucity of reliable surveillance data [48].

Recently, there has been an increase in the number of adolescent and young adults being diagnosed in some high-income countries, likely due to decreased vaccination uptake rates and a change in the vaccine itself. It is thought that the acellular vaccine currently used appears to have a short-lived immunity leading to increased infection rates [49]. Macrolide antibiotics are given to affected individuals to prevent further spread of pertussis [49].

Diphtheria presents as an acute infectious disease affecting the upper pulmonary tract caused by toxins produced by the bacterium. Characteristic features are membranous pharyngitis with fever, enlarged anterior cervical lymph nodes and edema of the soft tissues of the neck [50]. This manifestation may lead to airway obstruction. The bacterium has a short incubation period but untreated individuals may remain infectious for up to 4 weeks. Both pertussis and diphtheria are potentially fatal but easily preventable.

### Leptospirosis

Leptospirosis is contracted either through direct contact with infected animal urine, or urine-contaminated water [10]. It can be found worldwide, but is predominantly found in tropical and subtropical countries (during rainy season). Those taking part in water sports are most at risk (an outbreak was reported among canyoning participants in Martinique, France) [51]. Increased cases are also found in rural parts of a country where drinking water can be easily contaminated with the bacteria; for example, where wells have been poorly constructed [52]. Lately, there has been an increase in the number of cases noted in Europe, with 97 cases identified in the Netherlands in 2014 (4.6-fold increase in comparison to previous years). Dutch tourists reported 33 cases of leptospirosis, and most of these tourists acquired the disease in Thailand [53].

The clinical course of leptospirosis is variable, with symptoms from myalgia to fever with cough and shortness of breath. Weil’s disease is its severest form and may develop in 1–5% of cases. Patients progress from mild flu-like symptoms to signs of organ failure (jaundice and spontaneous bleeding) [54].

Where there is a high degree of clinical suspicion for leptospirosis, treatment should be instigated. Diagnosis is serological (via microscopic agglutination test [MAT] for antibodies or PCR urine antigen test) but it may take around 10 days after the onset of symptoms for these tests to become positive [10]. In early mild disease, penicillin and tetracycline antibiotics are effective [55]. Where the clinically more severe Weil’s disease is manifest, patients may require organ support, and there is little evidence that antibiotic use is of any benefit at this stage [56]. In 2014, there were a total of 76 confirmed cases of leptospirosis in England and Wales, of which 22 were acquired overseas; the majority of these were related to recreational water exposure [57].

### Tularemia

Recreational activities such as camping and visiting rural and agricultural areas lead to increased risk to exposure to the bacterium that causes tularemia [58]. Typically found in the Northern Hemisphere, the number of infected cases are underestimated and underreported [58]. Outbreaks have been reported on Martha’s Vineyard (Massachusetts, USA), Sweden and Finland in the last 15 years [58].

*Francisella tularensis* is the bacterium responsible for what is a potentially fatal multi-systemic disease. Reservoirs of transmission are via ticks, biting flies, aerosol, water exposure and food [58]. There are 4 identified subtypes and clinical presentation depends on biotype, method of transmission and port of entry. There are 6 major clinical presentations: ulceroglandular, glandular, oculoglandular, oropharyngeal, pneumonic and typhoidal [58]. Pulmonary features may consist of lobar pneumonia, pulmonary effusions, infiltrates and hilar lymphadenopathy. Mortality in untreated pneumonic tularemia can be up to 60% [59].

Early diagnosis and treatment is key to prognosis, but isolation of the bacterium is hazardous and time consuming and most laboratories do not accept samples for culture [60]. Agglutination and enzyme-linked immunosorbent assay (ELISA) tests are the diagnostic tests of choice [58]. Aminoglycosides are bactericidal against *F. tularensis* and WHO recommend a 10-day course [61].

As an aside, *F. tularensis* subspecies tularensis (Type A) is one of the most infectious pathogens known to man, and these properties have led to tularemia being identified as a potential weapon of bioterrorism [60].

### Melioidosis

In South East Asia and northern Australia, a particular Gram-negative organism called *B. pseudomallei* is responsible for an endemic of melioidosis disease [62]. Most commonly, the initial presentation is with low-grade fever and atypical pneumonia, rapidly progressing to multi-organ dysfunction. The mortality rate is very high, ranging from 14 to 40% in general and up to 80% with inappropriate antimicrobial therapy [9]. Difficulties in culturing the bacterium responsible mean that under-reporting is likely. The bacterium resides in soil and water with transmission occurring mainly from percutaneous inoculation and inhalation. Exposure is most likely to occur during the wet seasons in affected areas or after natural catastrophes such as tsunamis.

Sporadic cases of the infection are now being reported worldwide and manifestations of latent forms of the disease are appearing some tens of years after initial exposure [63]. As the number of international travellers continues to rise, particularly to more rural parts of South East Asia and northern Australia, this could lead to an increase in the number of travel-related melioidosis cases in the future.

Early recognition of melioidosis is essential as the bacterium is resistant to nearly all drugs given for community-acquired pneumonia [63]. Current treatment recommendations are ceftazidime or a carbapenem, followed by eradication therapy with trimethoprim and sulfamethoxazole for at least 3 months. There has been recent interest in vaccine development due to both public health and bioweapon concerns [64]. Mouse model vaccination has proved successful but to date there has been no testing on humans [64].

### Tuberculosis (TB)

TB is a commonly found disease in certain parts of the world. The risk of TB as a travel-related disease has not been well established. A study from Italy in 2005 concluded that over half of returned travellers with pneumonia or TB presented with a febrile illness but no pulmonary symptoms [65]. TB can be transmitted on airplanes; a flight from Baltimore to Honolulu showed 4 of 15 passengers had a positive tuberculin skin test after sitting in close proximity to a passenger with pulmonary TB [6]. An overall probability of infection is 1 in 1,000 when a symptomatic source is present in close proximity [6].

Risk is linked to longer-term travel to areas of higher prevalence and proximity to affected individuals. Those most at risk would be healthcare workers or individuals visiting relatives [66]. TB risk in long-term travellers (of greater than 3 months) is the same as the local population in high endemic areas. A Dutch study showed there was substantial risk to long-term travellers with an overall incidence rate of 3.5 per 1,000 person-months travelled; however, no-one in this study had received a Bacillus Calmette–Guérin (BCG) vaccine prior to travelling, therefore incidence amongst those who have been vaccinated is likely to be lower [66].

In recent years, multidrug-resistant TB (MDR-TB) and extensively drug-resistant TB (XDR-TB) have become increasingly important public health problems in many countries (Eastern Europe, parts of Asia and Southern Africa) [67]. The potential risk of transmission of particularly dangerous forms of TB requires renewed vigilance. Since 2006, WHO have been informed of several incidences of patients travelling (short-haul) by air with MDR-TB and XDR-TB. In these cases, only close family members have been later diagnosed with the disease, but a potential risk to other travellers is possible given the infectious nature of these strains of TB [67]. WHO have since published guidelines for TB and air travel to prevent the spread of the disease [67].

The main advice to clinicians is that the number of TB infections is increasing, as are MDR-TB and XDR-TB disease. The risks to most travellers in contracting TB are small but increase depending on vaccination status, length of travel, mode of travel and travel destination. If pulmonary symptoms persist, despite negative standard microscopy, culture and sensitivity testing, TB should be added to the differential diagnosis list and should be investigated appropriately.

## Pulmonary infections caused by fungi

Fungal infections in the absence of immunosuppression generally occur in the Americas, where the exact destination of the traveller plays an important role in distinguishing the fungus responsible.

### Histoplasmosis and coccidioidomycosis

Histoplasmosis should be considered in travellers returning from the Midwest and central states of North America, throughout Latin America and Central and Western Africa [68–70]. In 2013, approximately 300 cases were reported in the South West region of China, the first to be reported in Asia [68].

Coccidioidomycosis (Valley fever) has long been recognized as a travel-related mycosis. Endemic areas are in Southern California, Arizona and Central and South America [69]. In returned travellers when common causes for flu-like illness, non-productive cough and pleuritic chest pain have been ruled out but symptoms continue to persist, an underlying fungal infection should be considered as a differential diagnosis.

*Histoplasma capsulatum* is endemic in North and Latin America, while *H. dubiosii* is mainly found in Central and Western Africa [70]. The organism grows in soils enriched with bat and bird droppings, and human infection generally occurs after dust inhalation of disturbed soil. The incubation period is relatively short, and symptoms develop within 2 weeks of exposure [69]. Reticulonodular shadowing on chest radiograph is common, often with mediastinal lymphadenopathy. Detection of the histoplasma antibody in urine or serum is the most sensitive and widely used diagnostic method [70]. Detection of coccidioidomycosis (*Coccidioides immitis)* is similar but radiologically a cavitating pneumonia may be seen [69].

In healthy individuals, fungal infections are generally self-limiting. In those who are immunocompromised, and/or go on to develop severe infection with systemic complications, antifungals have been proven to be effective [70]. In contrast to histoplasmosis, coccidioidomycosis infection generally leads to acquired immunity therefore recurrence is unlikely [69].

### Pulmonary infections caused by helminths

Pulmonary symptoms such as wheeze, breathlessness and hemoptysis and an eosinophilia in a returned traveller should prompt investigations for helminths. These infections may present as Loeffler’s syndrome (a result of larval migration through the lungs) or tropical pulmonary eosinophilia (hypersensitivity to lymphatic filarial worms). Specific to schistosomiasis infections, Katayama syndrome should be suspected in travellers returning from Africa or South East Asia with fresh water exposure presenting with fever, eosinophilia, dry cough and urticarial rash. Other rare pulmonary presentation of helminths infections (such as paragonimus, toxocariasis and dirofilariasis) should be considered depending on travel destination and incubation period [71]. Treatment of these infections is generally straightforward once the causative helminths have been identified.

In 20% of hydatidosis (echinococcosis tapeworm) there are pulmonary infiltrations. The majority of cases are asymptomatic but following a leaking hydatid cyst in the liver, patients may present with pleuritic chest pain, breathlessness and cough; a ‘water lily’ sign may be seen on chest radiograph [72]. Surgery (aspiration) and anti-parasitic drugs are required to fully treat the infection.

## Conclusion

The clinical spectrum of pulmonary infections in the returned traveller is vast. History taking is paramount (exact dates, contacts, length of stay, place of travel and timings of onset of symptoms) and a high degree of suspicion early on in the patient’s management is vital. Most cases of pulmonary infections in travellers are mild and self-limiting but travel-specific pulmonary infections have high mortality rates if interventions are not prompt.

The main modes of transmission for pulmonary infections are by droplets or direct contact, hence the importance of implementing protective barriers and involving public health colleagues early to prevent further transmission. Advice is always available from online resources and specialist centers—as a clinician it is important to familiarize yourself with guidelines and protocols on travel-related infections. The WHO and Centers for Disease Control and Prevention (CDC) are continually updating guidelines on travel-related infections.

### Search strategy

We identified references for this review by searches in different databases (PubMed, Ovid MEDLINE, Embase and Cochrane) and references from relevant articles. The following MeSH headings were used: “pulmonary”, “respiratory”, “tropical disease” ‘travellers”, “travel”, “returning traveller”.

The references were all further assessed by reading the title and abstract. Only articles published in English were included. We further assessed the references of all selected publications. The final reference list was generated on the basis of relevance to the topics covered in this review.
